# Recombinant Type III Humanized Collagen Solution for Injection Promotes Skin Repair in Chinese Population: A Case Series

**DOI:** 10.1111/jocd.70226

**Published:** 2025-05-25

**Authors:** Xiaoguang Duan, Chunmei Ding, Jiewei Wu, Weiwei Bao, Yu Gao, Fei Liu, Wei Cai

**Affiliations:** ^1^ Department of Plastic Surgery Chingho Medical Center Shanghai China; ^2^ L'Oréal Research & Innovation China Advanced Research Shanghai China; ^3^ L'Oréal Dermatological Beauty China Skinceuticals Shanghai China; ^4^ Department of Plastic Surgery The Second Affiliated Hospital of Nanjing Medical University Nanjing China

**Keywords:** acne scarring, Chinese population, facial erythema, recombinant type III humanized collagen solution

## Abstract

**Background:**

Facial erythema and acne scarring are common skin conditions that significantly impair quality of life, necessitating effective treatment options, particularly in Asian populations.

**Aims:**

To evaluate the efficacy and safety of intradermal injections of recombinant type III humanized collagen (RhCol‐III) solution for alleviating facial erythema, improving skin texture associated with acne scarring, and evening out skin tone issues associated with post‐inflammatory hyperpigmentation (PIH) from acne while also searching for potential underlying anti‐inflammatory mechanisms of the RhCol‐III solution.

**Methods:**

This case series included six Chinese participants with Fitzpatrick phototypes III/IV, presenting with facial erythema and/or acne scarring. Participants received intradermal injections of 2 mg/mL RhCol‐III solution at baseline, 30‐ and 60‐ days post‐baseline. Clinical evaluations were conducted using VISIA photography and self‐assessed Global Aesthetic Improvement Scale (GAIS) scores. Additionally, in vitro testing was performed using the U937 cell line with lipopolysaccharide (LPS) induction to assess the anti‐inflammatory effects of the RhCol‐III solution on tumor necrosis factor‐alpha (TNF‐α) and interleukin‐6 (IL‐6) secretion.

**Results:**

All participants demonstrated a reduction in facial erythema post‐treatment, with significant improvements noted in the zygomatic area. The mean erythema area decreased by 49.3% after 90 days. One case with acne scarring showed visible improvement in scar appearance, skin tone, and texture. Moreover, 0.10 mg/mL RhCol‐III solution significantly reduced TNF‐α and IL‐6 levels in vitro by up to 47.75% and 44.96%, respectively.

**Conclusion:**

RhCol‐III solution injections effectively reduced facial erythema and have the potential to improve acne scarring via reduction in the extent of PIH in this series of Chinese cases. The treatment was well‐tolerated, with no significant adverse effects. These findings suggest RhCol‐III solution as a promising therapeutic option for skin repair and inflammation reduction.

## 1 Introduction

Facial erythema, characterized by redness and warmth, is a common outcome of skin injury. It can result from internal factors such as immunological responses [1, 2] or external causes like photodamage [3, 4]. Erythema, particularly from ultraviolet (UV) exposure, is a prominent sign of skin damage, often associated with inflammation, warmth, and tenderness [3, 4]. Chronic photodamage can lead to photoaging, marked by deep wrinkles, coarse texture, hyperpigmentation, and vascular changes [4]. Although the exact pathophysiology remains unclear, current theories suggest that erythema involves an exaggerated innate immune response (induction of pro‐inflammatory markers, like IL‐6 or TNF‐α), neurovascular degeneration, nitric oxide production, and cathelicidin peptides, all contributing to vasodilation and inflammation [5]. Impairment of the skin's barrier function and reduced antioxidants can exacerbate the condition [5]. A 2021 multicenter study in China reported a 2.4% prevalence of weekly facial erythema, with rates as high as 3.2% in northern regions [6]. This skin condition significantly impacts mental well‐being, often leading to social anxiety and even affecting potential employment opportunities [7].

Post‐inflammatory erythema (PIE) may occur as a sequela to acne vulgaris in Fitzpatrick type I–II skin, while PIH is a common sequela in Fitzpatrick type III–V skin [8]. Some degree of post‐acne scarring, including atrophic, rolling, boxcar, and ice‐pick scars, is common in many patients, owing to an altered wound healing response to inflammation and collagen loss at the lesion site [9]. These scars not only pose therapeutic challenges but also contribute to significant psychological distress, leading to decreased self‐esteem, anxiety, and depression [10]. The visible nature of facial scars amplifies these psychosocial effects, making effective treatment essential for both physical and mental health [10].

Various treatments for erythema and acne scars exist, including light‐based therapies such as lasers and intense pulsed light. However, these methods carry the risk of side effects like PIH, particularly in Fitzpatrick type III–V skin [8, 11]. Topical treatments aimed at repairing the skin barrier, restoring antioxidants, and using medications like vasoconstrictors for erythema [5] and retinoic acid for acne scarring [12] are also common, though these approaches require extended treatment times and strict adherence. Injectable treatments, including neuromodulators for erythema [5] and hyaluronic acid for atrophic scars [13], offer another option but are often limited by challenges in precise control of filling volume, which can result in side effects such as raised bumps.

Several studies have found that recombinant type III humanized collagen injection may have the potential to treat signs and symptoms of photoaging through stimulating skin repair and extracellular matrix remodeling [14, 15]. In this study, we aim to further explore the use of RhCol‐III solution injection in reducing skin erythema, improving skin texture associated with acne scarring, and evening out skin tone issues associated with PIH from acne while also searching for potential underlying anti‐inflammatory mechanisms. Herein, we present a case series demonstrating the clinical potential of this treatment, supported by an in vitro study highlighting its anti‐inflammatory effects.

## 2 Methods

### 2.1 Study Participants

This case series included six participants (three males and three females) aged between 30 and 43 years, with a mean age of 35.5 ± 5.2 years (Table 1). All participants were of Han Chinese ethnicity (100%) with Fitzpatrick phototypes III or IV. Three patients presented with mixed skin type (oily and dry, 50%) and three patients presented with oily skin type (50%). Acne scarring was a major skin quality issue for five participants (83%), while four participants (67%) reported issues with inflammation and erythema. Despite varying degrees of daily sun exposure and skincare routines, all participants (100%) reported consistent sunscreen use.

**TABLE 1:** Baseline data and clinical evaluation of six cases treated with RhCol‐III solution injection.

Case	Age	Sex	Skin type	Skincare issues prior to treatment	PMH: sun exposure and protection	PMH: skincare routine	PMH: aesthetic procedures	Best results achieved/duration for effect to be achieved	Improvements achieved with RhCol‐III	GAIS score
1	41	M	Mixed	Acne scarring, wrinkles, enlarged pores	Barely any sun exposure, excellent sunscreen adherence	Simple skincare routine	IPL procedure 3 months prior, RF procedure 6 months prior	After 1st injection/within 1 week (6 days)	Reduction in pore size, improvement in skin tone evenness	1
2	34	F	Mixed	Acne scarring, inflammation, enlarged pores	Sun exposure moderate, excellent sunscreen adherence	Moderately complex skincare routine	IPL procedure 6 months prior	After 2nd injection/within 1 week (3 days)	Reduction in inflammation and extent of erythema, improvement in radiance, firmness	1
3	30	M	Oily	Acne scarring, inflammation, enlarged pores	Sun exposure moderate, excellent sunscreen adherence	Moderately complex skincare routine	IPL procedure unknown date	After 1st injection/within 2 weeks	Reduction in inflammation and extent of erythema	2
4	32	F	Oily	Acne scarring, uneven skin tone, enlarged pores, hyperpigmentation	Sun exposure minimal, excellent sunscreen adherence	Moderately complex skincare routine	NAFL procedure half a year prior	After 3rd injection/within 2 weeks	Improvement in skin tone evenness and skin surface evenness, reduction in pore size	1
5	43	F	Mixed	Wrinkles, inflammation, uneven skin tone, enlarged pores, skin laxity, hyperpigmentation	Barely any sun exposure, excellent sunscreen adherence	Complex skincare routine	IPL procedure 3 months prior	After 1st injection/within 2 weeks	Reduction in inflammation and extent of erythema	1
6	33	M	Oily	Acne scarring, wrinkles, inflammation, uneven skin tone, enlarged pores, skin laxity, hyperpigmentation	Barely any sun exposure, excellent sunscreen adherence	Moderately complex skincare routine	laser procedure unknown date	After 2nd injection/within 4 weeks	Improvement in radiance, skin tone evenness	2

*Note:* Age refers to age prior to receiving RhCol‐III solution injection.

Abbreviations: F, female; GAIS, global Aesthetic Improvement Scale; IPL, intensive‐pulse light therapy; M, male; NAFL, non‐ablative fractional laser therapy; PMH, past medical history; RF, radiofrequency therapy; RhCol‐III, recombinant type III humanized collagen.

Cases were eligible for inclusion if they met either of the following criteria:

- Aged between 25 and 65 years old.
- Presentation with at least one of the following: acne scarring in a stable period, inflammation or erythema, and sensitive skin.
- Voluntarily consented to receive intradermal injections of RhCol‐III solution after being informed of all associated risks.

Cases were excluded from the series if they met either of the following conditions:

- Presence of signs of infection, open wounds, or active acne on the facial skin.
- Recent treatment with lasers, chemical peels, or any other facial procedures that could impact the dermal layer within 3 months prior to the study.
- Recent treatment with injectable dermatological or cosmetic agents (such as botulinum toxin or hyaluronic acid) on the face within 6 months of enrollment.
- Menstruation, pregnancy, or lactation.
- Known allergies to collagen products, RhCol‐III solution injection ingredients, or local anesthetics.
- History of autoimmune diseases, use of immunosuppressive medications, coagulative disorders, anticoagulant therapy, or severe heart or kidney dysfunction.

The case series was conducted following the principles of the World Medical Association Declaration of Helsinki. Written informed consent for the use of participant photographs in publications of study results was obtained from all participants prior to taking part in the study. Participant privacy rights were protected in accordance with ethical guidelines.

### 2.2 Administration Technique of RhCol‐III Solution Injection

**FIGURE 1:**
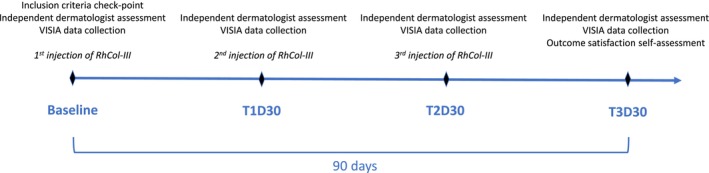
Timeline of RhCol‐III solution injection and treatment evaluation. RhCol‐III, recombinant type III humanized collagen; T1D30, 30 days post‐first RhCol‐III solution injection; T2D30, 30 days after second RhCol‐III solution injection; T3D30, 30 days after third RhCol‐III solution injection.

Recombinant type III humanized collagen solution for injection (RhCol‐III; Skinceuticals COLLAGEN‐III REGEN; manufacturer: Shanxi Jinbo Biomedical Co. Ltd.; Taiyuan, Shanxi, PR China; medical device registration: 20233131245) was administered at baseline, 30‐ and 60‐ days post‐baseline by a single investigator. Skin changes and patient satisfaction were assessed through clinical and instrumental evaluations (Figure 1).

Before injection, the skin was cleansed, and topical anesthesia (lidocaine and prilocaine) was applied for 30 min, followed by skin disinfection with iodine. A total of 8 mL of 2 mg/mL RhCol‐III solution was injected into the facial dermis of each patient. Using a 34‐gauge, 4 mm needle, 4 mL was injected evenly into the zygomatic, forehead, and periorbital areas. The remaining 4 mL was injected using an electronic injector with parameters set at 0.025 mL per injection, slow speed, 10% negative pressure suction, and 80% retraction force. Injection depths were 0.8 to 1.2 mm for the cheeks and 0.6 to 0.8 mm for the forehead and periorbital areas.

Post‐treatment, participants were instructed to apply collagen or hyaluronate masks once or twice daily, followed by a moisturizer after 3 days. Physical sun‐protection methods, such as hats, protective clothing etc., were advised immediately after treatment, and participants were recommended to switch to mild sunscreens after 3 days. Lifestyle guidance included adequate sleep, reduced alcohol intake, minimized sun exposure, and moderate exercise. Participants did not receive any concurrent aesthetic medical interventions, including but not limited to energy‐based device treatment, injectables, chemical peels, etc.

### 2.3 Outcome Evaluation of RhCol‐III Solution Injection

#### 2.3.1 Erythema and Acne Scarring

High‐resolution VISIA (Canfield Imaging Systems) photographs were taken at baseline and 30‐, 60‐, and 90‐ days post‐baseline. Quantitative changes in erythema areas, skin tone evenness, and skin smoothness were calculated using Image‐Pro Plus 7.0 image analysis software [16, 17].

#### 2.3.2 Patient Satisfaction

Self‐assessed Global Aesthetic Improvement Scale (GAIS) scores were collected from all participants after treatment to assess satisfaction with treatment outcome.

### 2.4 In Vitro Testing of the Effect of RhCol‐III Solution on Cellular Inflammation Markers

An in vitro test was conducted to explore RhCol‐III solution's anti‐inflammatory potential, using the U937 cell line (ATCC, Cat#CRL‐1593.2). U937 cells were cultured in RPMI 1640 medium (Gibco, Cat#31800‐022) containing 10% fetal bovine serum (Gibco, Cat#10099141) and incubated at 37°C with 90% humidity and 5% CO_2_. Cells were then harvested and seeded at a density of 5.0 × 10^5^/well in 12‐well plates and cultured for 24 hours in 2 mL RPMI 1640 medium with or without varying concentrations of RhCol‐III solution and dexamethasone. After 24 hours of incubation, media containing varying concentrations of RhCol‐III solution and 2 μg/mL LPS (Sigma, Cat#L2880) were changed to the test groups. The model control (M) group received 2 μg/mL LPS without RhCol‐III solution, while the positive control (PC) group received media containing dexamethasone and LPS, and the no treatment (NT) group received media only.

After another 24‐hours culture period, the supernatant was collected and stored at −80°C. Cytokine levels were measured using an ELISA kit, and TNF‐α and IL‐6 levels were expressed relative to the model group (100%).

## 3 Results

### 3.1 Effect of RhCol‐III Solution Injection on Facial Erythema

The majority of cases (5/6, 83%) reported significant improvements in erythema after the first or second injection of RhCol‐III solution. Similarly, five cases (83%) also indicated that the maximum effect was observed within 1–2 weeks postinjection. Four cases were very satisfied with the outcome (GAIS score = 1), while two cases reported being satisfied (GAIS score = 2).

**FIGURE 2:**
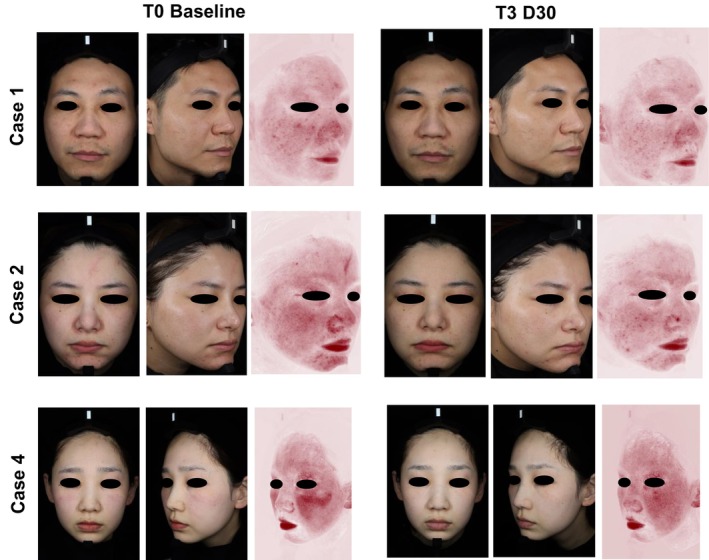
Frontal and oblique VISIA photographs of three representative cases before and after treatment with intradermal injection of RhCol‐III solution, including red areas. The reduction in erythema area and improvement in skin tone observed 3 months following the initial RhCol‐III solution injection is depicted. RhCol‐III, recombinant type III humanized collagen; T3D30, 30 days after third RhCol‐III solution injection.

**FIGURE 3:**
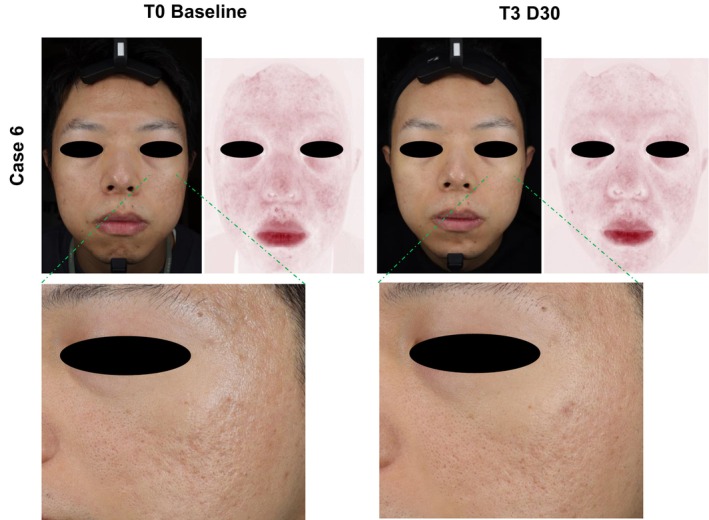
VISIA photographs of a 33‐year‐old male (Case 6) taken before and 3 months after the initial RhCol‐III solution treatment, including red areas and a close‐up onto zygomatic area on the left side of the face (extensive acne scarring zone). The reduction in acne scarring area observed 3 months following the initial RhCol‐III solution injection is depicted. RhCol‐III, recombinant type III humanized collagen; T3D30, 30 days after third RhCol‐III solution injection.

A reduction in erythema was noted in all six cases, as demonstrated in the VISIA photographs taken before and after treatment (Figures 2 and 3). The most significant reduction was observed in the zygomatic area (bilateral cheeks). Based on VISIA image analysis, the mean erythema area decreased by 49.3% after 90 days. Improvements in erythema were noted as early as 30 days post‐injection in five cases and persisted throughout the 90‐day study period in four cases (Table 2).

**TABLE 2:** Erythema area changes in six cases that presented with facial erythema and were treated with RhCol‐III solution injection.

Case	Baseline	T1D30	T2D30	T3D30
1	39.0	15.0 (−61.5%)	8.0 (−79.5%)	14.9 (−61.8%)
2	37.0	42.0 (+13.5%)	14.0 (−62.2%)	21.2 (−42.7%)
3	21.2	11.4 (−46.2%)	26.4 (+24.5%)	11.0 (−48.1%)
4	28.0	24.1 (−13.9%)	13.7 (−51.1%)	19.5 (−30.4%)
5	27.9	15.6 (−44.1%)	10.0 (−64.2%)	11.0 (−60.6%)
6	32.1	27.0 (−15.9%)	19.0 (−40.8%)	16.3 (−49.2%)
Mean	30.9	22.5 (−27.1%)	15.2 (−50.8%)	15.7 (−49.3%)
SD	6.6	11.2	6.7	4.2

*Note:* Units: pixels. Percentage values in brackets refer to the rate of improvement compared to baseline. A lower value indicates an improvement in erythema.

Abbreviations: RhCol‐III, recombinant type III humanized collagen; SD, standard deviation; T1D30, 30 days post‐first RhCol‐III solution injection; T2D30, 30 days post‐second RhCol‐III solution injection; T3D30, 30 days post‐third RhCol‐III solution injection.

No significant adverse events were reported during the study period. All participants tolerated the treatment well, with no incidents requiring medical intervention.

### 3.2 Effect of RhCol‐III Solution Injection on Facial Acne Scarring

Imaging analysis of VISIA photographs revealed that all cases that presented with acne scarring (five out of five) had consistent improvement in skin tone evenness over time (Table 3), as well as skin smoothness at various time points post‐baseline (Table 4). As a compensatory response to inflammation, such as acne vulgaris, skin may produce more melanin and lead to post‐inflammatory hyperpigmentation (PIH) and thus lead to uneven skin tone. There was a noteworthy improvement in skin tone evenness in the five cases that presented with acne scarring at baseline, with mean scores of skin tone unevenness continuously decreasing by 20.5%, 26.2%, and 40.2% at 30‐, 60‐, and 90‐days post‐baseline, respectively (Table 3). This improvement was observed as early as 30 days post‐injection in three of the cases. Owing to the long‐term presence of the scars in the five participants, skin smoothness improvements were more challenging to achieve. This indicator only improved by 9.54% at 60 days post‐baseline, meaning that several rounds of RhCol‐III solution injection may be required to enhance the effect on skin texture (Table 4). Four cases had shown an improvement in skin smoothness by 90 days post‐baseline.

**TABLE 3:** Skin tone evenness changes in five cases that presented with post‐inflammatory hyperpigmentation from acne scarring and were treated with RhCol‐III solution injection.

Case	Baseline	T1D30	T2D30	T3D30
1	2.79	1.59 (−43.0%)	1.58 (−43.4%)	1.36 (−51.3%)
2	2.22	2.38 (+7.20%)	2.00 (−9.90%)	1.77 (−20.3%)
3	4.01	2.89 (−27.9%)	3.02 (−24.7%)	2.27 (−43.4%)
4	3.31	2.31 (−30.2%)	2.09 (−36.9%)	1.44 (−56.5%)
6	1.64	1.94 (+18.3%)	1.62 (−1.20%)	1.52 (−7.3%)
Mean	2.79	2.22 (−20.5%)	2.06 (−26.2%)	1.67 (−40.2%)
SD	0.83	0.49	0.58	0.37

*Note:* Index of skin tone evenness as calculated with Wu and Tanaka method [17]. Percentage values in brackets refer to the rate of improvement compared to the baseline. A lower index value indicates an improvement in skin tone evenness. Case 5 was not included, since it did not present with acne scarring at baseline upon clinical evaluation by a dermatologist.

Abbreviations: RhCol‐III, recombinant type III humanized collagen; SD, standard deviation; T1D30, 30 days post‐first RhCol‐III solution injection; T2D30, 30 days post‐second RhCol‐III solution injection; T3D30, 30 days post‐third RhCol‐III solution injection.

**TABLE 4:** Skin smoothness changes in five cases that presented with post‐inflammatory hyperpigmentation from acne scarring and were treated with RhCol‐III solution injection.

Case	Baseline	T1D30	T2D30	T3D30
1	18.97	20.04 (+5.64%)	15.09 (−20.45%)	17.36 (−8.49%)
2	16.04	17.33 (+8.04%)	16.09 (+0.31%)	15.99 (−0.31%)
3	18.18	17.33 (−4.68%)	16.09 (−11.50%)	16.84 (−7.37%)
4	12.34	12.59 (+2.03%)	12.17 (−1.38%)	14.17 (+14.83%)
6	19.51	18.84 (−3.43%)	17.49 (−10.35%)	16.59 (−14.97%)
Mean	17.01	17.23 (+1.28%)	15.38 (−9.54%)	16.19 (−4.81%)
SD	2.92	2.83	1.99	1.23

*Note:* Index of skin smoothness as calculated with Wu and Tanaka method [17]. Percentage values in brackets refer to the rate of improvement compared to baseline. A lower index value indicates an improvement in skin smoothness. Case 5 was not included, since it did not present with acne scarring at baseline upon clinical evaluation by a dermatologist.

Abbreviations: RhCol‐III, recombinant type III humanized collagen; SD, standard deviation; T1D30, 30 days post‐first RhCol‐III solution injection; T2D30, 30 days post‐second RhCol‐III solution injection; T3D30, 30 days post‐third RhCol‐III solution injection.

Of the five cases presenting with acne scarring, one (Case 6, male, 33 years old) showed marked visible improvement after RhCol‐III solution injection, as seen in VISIA photographs (Figure 3). Case 6 had been dealing with facial acne scarring for over 15 years, exhibiting scarring predominantly on both sides of his cheeks, around the cheekbones, and in the temporal regions. The majority of the scars were rolling scars, while also having some boxcar and icepick scars. Previously, the participant had undergone three sessions of CO_2_ fractional laser therapy, with different extents of improvement with concomitant PIH, which persisted prior to enrollment in our case series. Following our intervention, rolling scars appeared shallower, leading to smoother skin texture, particularly in the cheek and temple areas. While boxcar and icepick scars were more resistant to treatment, improvements were observed in terms of scar sharpness and depth. The treatment also resulted in a notable reduction in redness and hyperpigmentation, contributing to a more uniform skin tone. Case 6 exhibited a significant reduction in erythema area by 49.2% 3 months post‐injection, and the participant's GAIS score was 2, indicating satisfaction with the outcome.

### 3.3 Effect of RhCol‐III Solution Treatment on TNF‐Alpha and IL‐6 Secretion in LPS‐Induced U937 Cell Line

**FIGURE 4:**
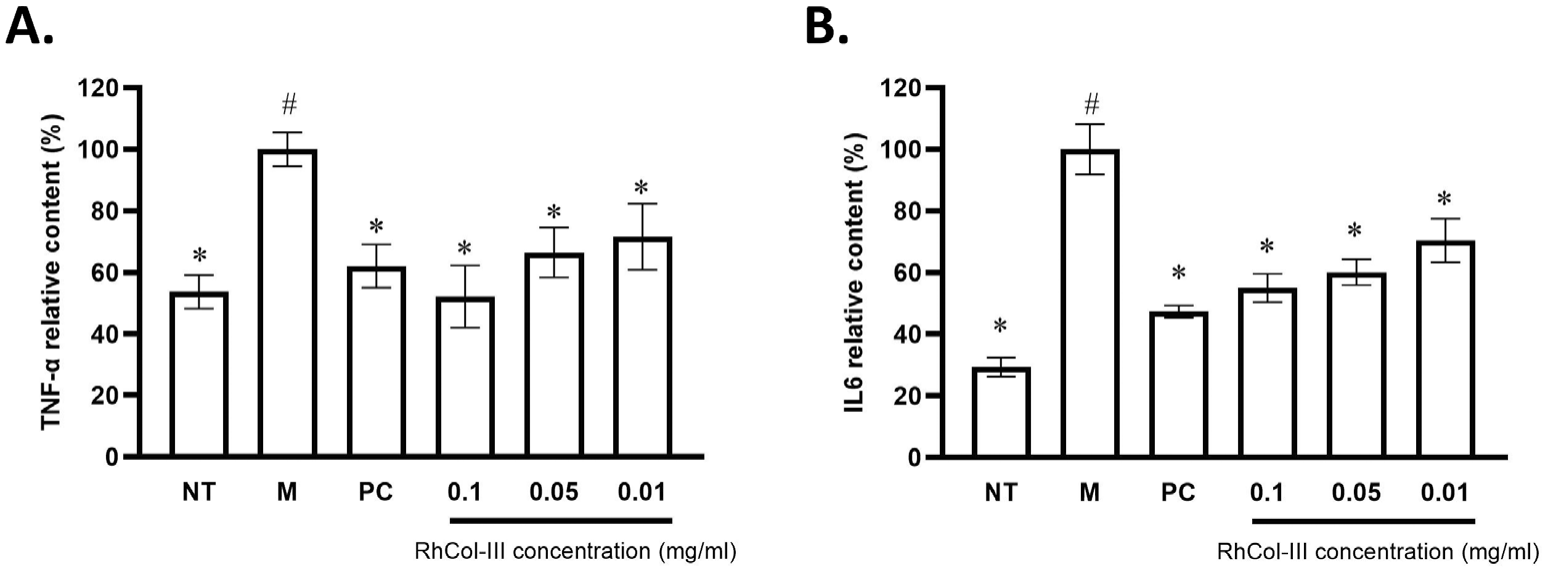
Effect of RhCol‐III solution intervention on U937 cell line secretion of TNF‐alpha and IL‐6 (a, b). M, model control, LPS only; NT, no treatment, RPMI 1640 medium; PC, positive control, medium with LPS and dexamethasone; RhCol‐III, recombinant type III humanized collagen solution at three concentrations, 0.10, 0.05, and 0.01 mg/mL. Statistical analysis was conducted using *t*‐test. A *p*‐value of less than 0.05 was considered statistically significant (*p < 0.05 compared to M, ^#^ *p* < 0.05 compared to NT).

In the model control group (M), LPS treatment significantly induced the secretion of the inflammatory factor TNF‐α, compared to the no treatment group (NT) (*p* < 0.05). The positive control group demonstrated a significant decrease in TNF‐α levels (*p* < 0.05), confirming successful modeling. In comparison to the model control group, RhCol‐III solution treatment led to a significant reduction in TNF‐α levels by 47.75%, 33.45%, and 28.38% at concentrations of 0.10, 0.05, and 0.01 mg/mL, respectively (*p* < 0.05, Figure 4A).

Similarly, IL‐6 levels were significantly higher in the model control group compared to the no treatment group (*p* < 0.05). The positive control group exhibited a significant decrease in IL‐6 levels (*p* < 0.05). Compared to the model control group, RhCol‐III solution treatment reduced IL‐6 levels by 44.96%, 39.86%, and 29.53% at concentrations of 0.10, 0.05, and 0.01 mg/mL, respectively, with significant differences (*p* < 0.05, Figure 4B).

## 4 Discussion

This study demonstrates that recombinant type III humanized collagen (RhCol‐III) solution injections effectively reduced facial erythema in all six cases at 90 days post‐baseline, improved skin tone evenness in all five cases of acne scarring at 60 days post‐baseline, while also improving skin texture in four cases of acne scarring at 60 days post‐baseline. The once‐a‐month injection schedule led to accumulating positive effects over the 90‐day period. Additionally, in vitro testing revealed that RhCol‐III solution significantly decreased TNF‐α and IL‐6 secretion, indicating its potential anti‐inflammatory capacity. The observed effects of RhCol‐III solution in the six cases that we presented, along with the in vitro results, add to the existing literature on the role of collagen in improving overall skin quality [18, 19]. Herein, our findings support the role of collagen in skin repair, highlighting it as a promising therapeutic option for addressing skin inflammation.

A combination of manual and electronic injection was implemented in our study, as manual injection allowed the investigator to enhance the effects of the treatment to a localized area of acne scarring. All cases that presented with PIH and skin texture issues from acne scarring on the face exhibited measurable improvements in skin tone evenness and smoothness; however, one case had visibly marked improvement in these parameters (Case 6). Genetic factors play a critical role in individual responses to dermatological treatments, including collagen‐based therapies. Variations in genes related to collagen synthesis and degradation, such as those encoding for matrix metalloproteinases (MMPs), enzymes responsible for collagen degradation, and tissue inhibitors of metalloproteinases (TIMPs), can influence the effectiveness of treatments aimed at improving skin quality. Polymorphisms in genes like MMP‐1 and MMP‐3 have been linked to altered collagen breakdown, impacting skin's structural integrity and response to therapies targeting collagen regeneration. Additionally, genetic predispositions influencing inflammation, such as variations in interleukin genes (e.g., IL‐6, IL‐1β), may affect the degree of erythema and scarring, leading to differing outcomes among patients receiving the same treatment [20].

Yang et al. proposed that the anti‐inflammatory mechanism of collagen operates through multiple pathways [21]. First, the direct replenishment of collagen within the dermal extracellular matrix (ECM) creates a micro‐filling effect, which enhances mechanical interactions between fibroblasts and the surrounding ECM. Increased mechanical tension promotes fibroblast function, reducing the likelihood of fibroblast senescence and maintaining ECM homeostasis. In addition, collagen supplementation appears to inhibit TGF‐β signaling, a key regulator in the fibrotic and inflammatory responses. By suppressing TGF‐β, collagen may help to downregulate pro‐inflammatory pathways. Simultaneously, the presence of exogenous collagen has been shown to decrease the activity of MMPs. This reduction in MMP activity results in an increased collagen presence within the ECM, further stabilizing the dermal structure. The stabilization of the ECM through collagen enrichment fosters an environment conducive to tissue repair and regeneration, which in turn diminishes the activation of pro‐inflammatory cytokines, such as TNF‐α and IL‐6. Consequently, the reduction of cytokine levels alleviates the chronic inflammatory response commonly seen in aged or damaged skin.

Our findings in the in vitro test are similar to a study by Brandao‐Rangel et al. which showed that hydrolyzed type III collagen is able to inhibit inflammatory response induced by LPS, suggesting that collagen is useful for maintaining immune integrity of skin [22]. IL‐6 and TNF‐α play crucial roles in skin inflammation and the regulation of collagen synthesis. IL‐6 is involved in the activation and recruitment of immune cells, contributing to chronic inflammation in conditions like psoriasis and atopic dermatitis. It also affects fibroblast activity, promoting collagen degradation and impairing tissue repair when dysregulated [23]. Similarly, TNF‐α is a key driver of inflammatory responses in the skin, exacerbating collagen breakdown through the activation of MMPs. Excessive TNF‐α levels can lead to collagen depletion, hindering the skin's structural integrity and delaying wound healing [24].

All in all, as a novel modality in medical aesthetics for Fitzpatrick type III–V skin, collagen injection has the potential to fulfill aesthetic needs not limited to skin repair and anti‐inflammation following further study. Collagen injection has a faster onset of action compared to topical skincare products, as it can directly replenish collagen content. Compared to chemical peels and light‐based device therapy, collagen injection can impact dermal thickness and ECM composition by directly supplementing type III collagen and regulating type I collagen generation by stimulating fibroblasts. Other energy‐based device therapy, such as radiofrequency therapy, which stimulates neocollagenesis through thermal effects, may cause some side effects, such as persistent erythema. Moreover, opposed to hyaluronic acid fillers, it can directly boost collagen content in the skin. The safety of RhCol‐III solution was assessed, and no adverse reactions were reported by participants throughout the treatment and follow‐up period.

The primary limitation of this study is its case series design, which included a small sample size of only six participants. While the findings are promising, a larger study with a split‐face design is needed to more robustly compare the effects of RhCol‐III solution treatment against control areas. Additionally, conducting a blinded evaluation would minimize bias and provide more objective assessments of treatment efficacy. A study with a larger sample size and a split‐face control design would also help us to adjust for confounding factors, such as by including stratification by age or skin type. Future research should explore combining recombinant type III humanized collagen injections with other medical aesthetic modalities, such as ultrasound, laser treatments, or intense pulsed light (IPL), to determine whether these combinations can enhance therapeutic outcomes and improve skin quality further. To increase standardization among future studies, some clinical grading scales, such as the Postinflammatory Dyspigmentation Area and Severity Index (PIDASI) or its modified version, Postinflammatory Hyperpigmentation Area and Severity Index (PIHASI) could be used to assess skin tone changes in a more objective manner [25], while also using more advanced erythema quantification techniques [26].

## 5 Conclusion

In this case series, we have demonstrated that dermal injection of recombinant type III humanized collagen solution can safely promote skin repair in Chinese populations, particularly in addressing erythema and acne scarring via improvement in skin tone and texture. The treatment showed favorable outcomes with no significant adverse effects. Initial in vitro testing demonstrated the ability of RhCol‐III solution to reduce inflammation by suppressing pro‐inflammatory markers. However, further research, such as cohort studies or randomized controlled trials, is necessary to validate these findings and explore the broader potential of this therapy for skin rejuvenation and repair.

## Author Contributions

W.C., X.D., F.L., and Y.G. designed and planned the study; W.C. and X.D. performed the aesthetic procedures and clinical evaluation; C.D. conducted in vitro testing; C.D., J.W., and W.B. performed data acquisition, analysis and presentation; W.C., C.D., and J.W. wrote the initial version of the manuscript; all authors reviewed the manuscript; W.C., F.L., and Y.G. prepared the final version of the manuscript.

## Consent

Informed consent was obtained from all individual participants included in the study.

## Conflicts of Interest

The authors declare no conflicts of interest.

## Data Availability

The data that support the findings of this study are available on request from the corresponding author. The data are not publicly available due to privacy or ethical restrictions.
